# An Adjustable Gas-Mixing Device to Increase Feasibility of *In Vitro* Culture of *Plasmodium falciparum* Parasites in the Field

**DOI:** 10.1371/journal.pone.0090928

**Published:** 2014-03-06

**Authors:** Amy K. Bei, Saurabh D. Patel, Sarah K. Volkman, Ambroise D. Ahouidi, Daouda Ndiaye, Souleymane Mboup, Dyann F. Wirth

**Affiliations:** 1 Department of Immunology & Infectious Diseases, Harvard School of Public Health, Boston, Massachusetts, United States of America; 2 Laboratory of Bacteriology and Virology, Faculty of Medicine and Pharmacy, Cheikh Anta Diop University, Dakar, Senegal; 3 Division of Gastroenterology, Hepatology and Nutrition, Boston Children’s Hospital, Boston, Massachusetts, United States of America; 4 Broad Institute of MIT and Harvard, Cambridge, Massachusetts, United States of America; 5 School for Nursing and Health Sciences, Simmons College, Boston, Massachusetts, United States of America; 6 Laboratory of Parasitology and Mycology, Faculty of Medicine and Pharmacy, Cheikh Anta Diop University, Dakar, Senegal; Agency for Science, Technology and Research - Singapore Immunology Network, Singapore

## Abstract

A challenge to conducting high-impact and reproducible studies of the mechanisms of *P. falciparum* drug resistance, invasion, virulence, and immunity is the lack of robust and sustainable *in vitro* culture in the field. While the technology exists and is routinely utilized in developed countries, various factors–from cost, to supply, to quality–make it hard to implement in malaria endemic countries. Here, we design and rigorously evaluate an adjustable gas-mixing device for the *in vitro* culture of *P. falciparum* parasites in the field to circumvent this challenge. The device accurately replicates the gas concentrations needed to culture laboratory isolates, short-term adapted field isolates, cryopreserved previously non-adapted isolates, as well as to adapt *ex vivo* isolates to *in vitro* culture in the field. We also show an advantage over existing alternatives both in cost and in supply. Furthermore, the adjustable nature of the device makes it an ideal tool for many applications in which varied gas concentrations could be critical to culture success. This adjustable gas-mixing device will dramatically improve the feasibility of *in vitro* culture of *Plasmodium falciparum* parasites in malaria endemic countries given its numerous advantages.

## Introduction

*In vitro* culture of the *P. falciparum* parasite remains a major advance in malaria research [1], and has resulted in a greater understanding of parasite biology compared to other *Plasmodium species* without such long term, robust culture systems [2], [3]. Cryopreservation has allowed the transport of samples from malaria endemic countries to non-endemic countries that have the resources to perform sophisticated biological experiments. However, strains can fail adaptation after cryopreservation that would have succeeded if cultured *ex vivo* for longer in the field. While cryopreservation and culture adaptation have made performing experiments with patient samples possible, it is important to consider that many features that affect disease pathogenesis, such as variant expression of antigenic families, can change with culture adaptation [4], [5]. The ideal situation would be to have the ability to perform robust *ex vivo* as well as reproducible *in vitro* assays and improve the capacity for long term *in vitro* culture of malaria parasites in the field [6], bringing us closer to the ultimate goal of performing experiments as close as possible to the *in vivo* state of the parasite within the human host.

Studies that routinely utilize *ex vivo* or *in vitro* culture techniques for *P. falciparum* use pre-mixed gas combinations: 1–5% O_2,_ 5% CO_2_, N_2_ balance. While such gas mixtures are readily available and affordable in developed countries, they are not locally available in most malaria endemic countries, especially in Sub-Saharan Africa, which bears the preponderance of the disease burden. The alternative for African malaria researchers is to order mixed gas from abroad, which is 20–150 times more expensive than in developed countries, and can have long wait times for delivery. For example, our group in Senegal has waited more than a year from order to delivery of pre-mixed cylinders. The other alternative is to culture parasites for single-cycle *ex vivo* assays in a “candle jar” – a desiccator chamber in which a candle is lit and will self-extinguish when most of the oxygen is combusted [7]. Candle jars are simple, but labor intensive and require daily media changes in order to maintain the parasite growth through properly buffered pH [1], [8], [9]. Additionally, culturing by this method is not always optimal for long-term culture adaptation of field isolates [10], [11] or malaria parasites of different species [12]. The main advantage to mixed gas over the candle jar is the reproducibility of the gas concentrations obtained and the amenability to high throughput assays.

We sought to meet the challenge of reliable, sustainable, and cost-effective gas for malaria culture in the field by adapting a device commonly used to mix gases for welding purposes. While we are not the first to suggest a gas-mixing device for malaria endemic countries [13], this report is the first to rigorously test and validate such a device against the gold-standard of pre-mixed imported gas, for both laboratory adapted lines and field isolates, with gas concentration measurements. Additionally, we outline in detail the necessary steps to assemble, test, and use this gas-mixing device, which will enhance the feasibility of conducting robust *ex vivo* and *in vitro* culture and assays in the field, as close to the *in vivo* parasite biology as possible. We further suggest that use of such an adjustable device could have implications and applications for the culture adaptation of parasites representing different disease states of *P. falciparum* as well as other *Plasmodium* species which have proved refractory to culture adaptation efforts thus far.

## Results and Discussion

### Field Implementation Considerations

**Figure 1:**
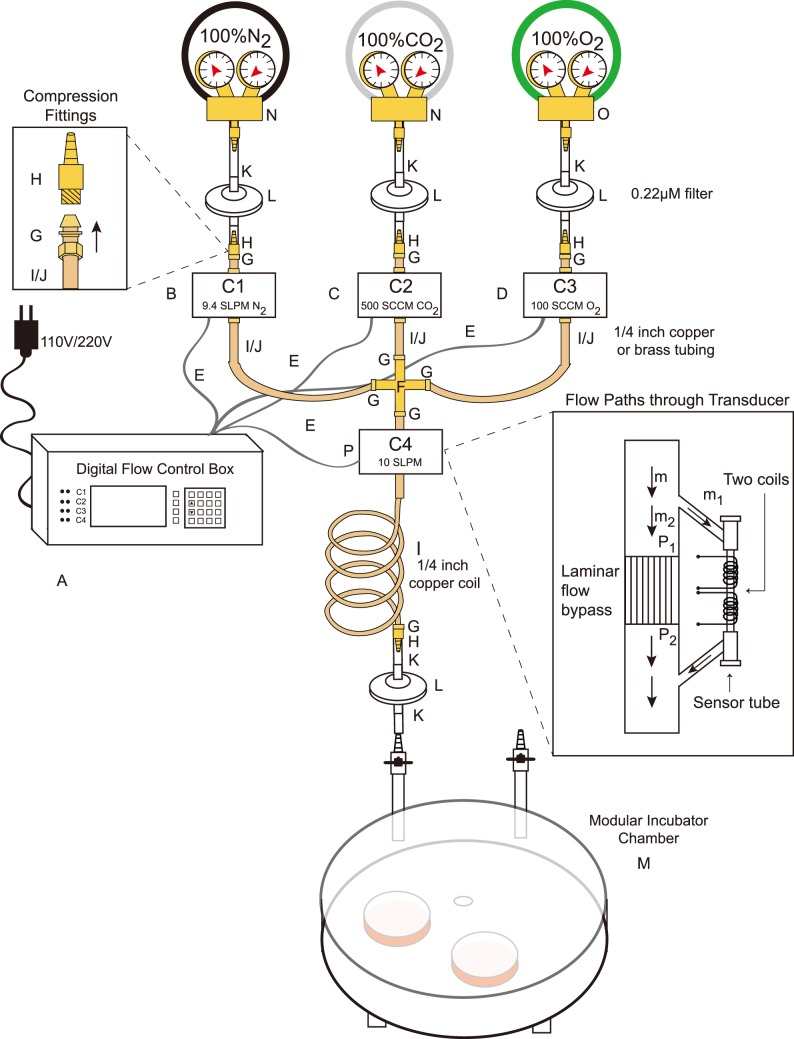
Diagram of digital flow control box with flow controllers. A diagram showing the set up of the digital control box and gas flow controllers. Inset diagrams the principle of the flow controls as adapted from the manual. Individual parts are detailed in Table 1 in addition to as follows: A: Model 954 Digital Flow Box, B: 840L Mass Flow Controller –10 SLPM (N_2_) capacity, C: 840L Mass Flow Controller –1000 SCCM (CO_2_) capacity, D: 840L Mass Flow Controller –1000 SCCM (O_2_) capacity, E: 840-CDCL (15 ft) Cable, F: Compression fitting Union Cross, G: Compression threaded adapter, H: Barbed fittings, I: Copper tubing, J: Brass tubing, K: Clear PVC tubing, L: Pall Acro 0.2 µm PTFE vent filter, M: Modular Incubator Chamber, N: N_2_/CO_2_ Gas regulator, O: O_2_ Gas regulator, P: (Optional) 840L Mass Flow Controller –1000 SCCM (Air). Gas flow rates shown for B, C, and D correspond to a total flow rate of 10 SLPM at the appropriate gas percentages for parasite culture (94% N_2_, 5% CO_2_, 1% O_2_).

In setting up and testing our gas-mixing device (Figure 1), a number of implementation challenges were identified. The first challenge is that of dust. As suggested to us by the company, the flow box and controllers are very robust, however are very sensitive to dust. Therefore, we installed Pall Acro 0.2 µm PTFE vent filters (Figure 1, Item L) at the output of each gas cylinder to protect each downstream sensor, and after the copper coil to protect the parasites in the incubator chamber. In-line filters are available for the ¼ inch copper or brass tubing as well (Table 1, Item Q) and we will use these going forward. We keep our box and sensors protected with dust covers to try to minimize dust accumulation as much as possible.

**Table 1:** Necessary supplies and useful accessories for assembling an adjustable gas mixer.

Item	Vendor	Catalog Number	Description (Use)	Number Needed	Figure 1 Code
Model 954 Digital Flow Box	Sierra Instruments	954-PS-V1	Control box to control the flow rates monitored by the 3 flow controller/sensors	1	A
840L Mass Flow Controller – (N_2_, 10 SLPM)	Sierra Instruments	840L-2-OV1-SV1-D-V1-S1-840L	Controlling the N_2_ Flow Rate	1	B
840L Mass Flow Controller - (CO_2,_ 1000 SCCM)	Sierra Instruments	840L-2-OV1-SV1-D-V1-S1-840L	Controlling the CO_2_ Flow Rate	1	C
840L Mass Flow Controller – (O_2_, 1000 SCCM)	Sierra Instruments	840L-2-OV1-SV1-D-V1-S1-840L	Controlling the O_2_ Flow Rate	1	D
840-CDCL (15 ft.) Cable to go to display box	Sierra Instruments	840-CDCL	Connecting the Flow controllers to the control box	3	E
Compression fitting Union Cross, 1/4 inch innerdiameter	Parker Hannifin	4ECR4-B	Connecting all 3 flow controllers to the cross enabling a single output	1	F
Compression threaded adapter, brass, 1/4″male NPT	Cole-Parmer	EW-31412-35	Connecting Flow Controllers to copper/brass tubing	16	G
Barbed fittings, NPT male pipe adapter, Brass, 1/4″NPT male to 3/8 inch tubing	Cole-Parmer	EW-30904-11	Connecting the Flow controllers to the gas cylinders via the clear PVC tubing	1 (5 pack)	H
Copper tubing 1/4 inch outer diameter	Hardware store		Connecting the Flow Box to the Flow Controllers and cross; making the mixing coil	15 feet	I
Brass tubing 1/4 inch outer diameter, 1 foot long	Hardware store		Connecting the Flow Box to the Flow Controllers and cross	12	J
Nalgene 180 Clear PVC Tubing (inner diameter3/8″, outer diameter 1/2″)	Thermo Scientific	8000-4120	Connecting the Flow controllers to the gas cylinders	50 feet	K
Pall Acro 50 0.2um PTFE vent filter	Pall Corporation	4251	Filtering the gas exiting the cylinders, filtering the gasentering the modular incubator chamber	4	L
Modular Incubator Chamber	Billups-Rothenberg	MIC-101	Incubator chamber for culturing parasites (2 PSI max input)	1 (at least)	M
100% N_2_ gas, medical grade	Local Gas Supplier		N_2_ gas source	1	
100% CO_2_ gas, medical grade	Local Gas Supplier		CO_2_ gas source	1	
100% O_2_ gas, medical grade	Local Gas Supplier		O_2_ gas source	1	
N_2_/CO_2_ gas regulator	Local Gas Supplier		Regulator for N_2_ and CO_2_ gas, do not exceed 25 psi (1.75 bar)	2	N
O_2_ gas regulator	Local Gas Supplier		Regulator for O_2_ gas, do not exceed 25 psi (1.75 bar)	1	O
Dräger X-am 5000 Gas Monitor	Dräger	4543749	Monitoring purity of gas in the cylinders, measuring the output gas percentages after mixing	1^a^	
Dräger Sensor XXS E O_2_	Dräger	6812211	Measuring the O_2_ percentage	1^a^	
Dräger Sensor XXS CO_2_	Dräger	6810889	Measuring the CO_2_ percentage	1^a^	
Dräger Calibration cradle	Dräger	8318752	Adapting the gas monitor to small space measurement (tubing connecting to incubator chamber)	1^a^	
USB DIRA with USB cable, communicationadapter infrared to USB	Dräger	8317409	Electronically recording gas levels for downstream analysis	1^a^	
840L Mass Flow Controller - 10 SLPM	Sierra Instruments	840L-2-OV1-SV1-D-V1-S1-840L	Checking the flow rates of each sensor, calibrated for Air	1^a^	P
840-CDCL (15 ft.) Cable to go to display box	Sierra Instruments	840-CDCL	Connecting the Flow controllers to the control box	1^a^	E
Copper Tubing Cutter	Hardware store		Cutting the copper tubing with an even “square” cut	1^a^	
Ultra-high efficiency 0.01 micron in line filters	Cole-Parmer	EW-02917-60	Preventing dust from damaging flow controllers	4^a^	

a Non-essential, but useful accessories.

Another important consideration is the frequency of power surges and/or outages in malaria endemic countries. To circumvent the challenge of power surges, we keep the gas-mixing device plugged into a voltage converter/surge protector and upon shutdown, we unplug the power cable from the digital flow box (Figure 1, Item A). To allow for uninterrupted use during power outages, one might envision adapting car batteries, solar powered generators, or other energy alternatives as are frequently employed to resource-poor settings. Converters exist which allow the 12 V DC battery of a car to run a 115 V AC battery, which is the energy input requirement needed to run the gas mixer.

**Figure 2:**
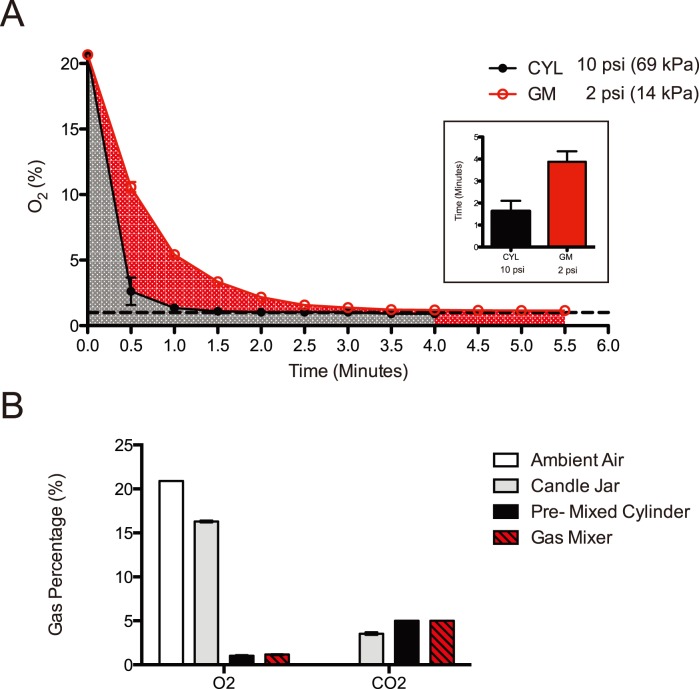
Gas concentration comparisons for field applicable culture methods. A. Graph of time and oxygen percentage as measured through modular incubator chamber output nozzle. Inset shows the time to reach 1% O_2_ for each method. Four independent experiments were performed and error bars represent standard deviation. B. Concentrations of CO_2_ and O_2_ for field applicable culture methods: Candle Jar, Pre-mixed gas cylinder (CYL), and gas-mixing (GM) device, compared to ambient atmospheric percentages. Three independent measurements were made (with five measurements for the gas-mixing device) and error bars represent standard deviation.

A third and very critical consideration for the success of our gas-mixing device is the quality of the input gas. To directly assess the gas quality, we found the Dräger X-am 5000 Gas Monitor to be an essential accessory (Table I, Useful accessories). On our first test of the gas mixer, we observed the oxygen levels were not reaching the desired 1.0%, but remained constant at 18.6%. We tested each gas cylinder independently, and discovered that the nitrogen gas which had been delivered was 17.6% O_2_, 0.6% CO_2_, and nitrogen balance–rather than 100% pure N_2_ because the company had supplied us with “industrial grade” nitrogen cylinder instead of “medical grade” nitrogen. When we obtained the correct “medical grade” nitrogen cylinder, the desired 0% O_2_, 0% CO_2,_ and 100% N_2_ was observed. Upon installing the medical grade nitrogen, our gas mixer gave the desired gas percentages (Figure 2). We anticipate that the gas quality may represent a substantial challenge in malaria endemic countries as without a Gas Monitor, there is no way to independently verify the gas quality prior to mixing, and gas percentages are not always tested by each company. This challenge can be overcome as long as “medical grade” gas is ordered because medical grade gas is usually held to a higher standard of quality control. In addition, the concentrations should be verified using a Gas Monitor.

### Effect of Gas Concentration on Parasite Processes

We first determined the time to optimal gas concentration for the gas-mixing (GM) device. Of note, the mass flow sensor used in the GM will deliver a consistent amount of gas over a broad temperature range, in contrast to volumetric gas mixers. The benefit of this type of sensor is that it accurately measures mass of gas regardless of temperature and pressure. Our flow controller was designed to have a maximum output pressure of 2 PSI (14 kPa) and 10 standard liters per minute (SLPM) at 70 degrees F and 1 atmosphere (14.5 PSIA absolute pressure) – in keeping with the maximum flow rate capacity of the modular incubator chamber (Billups-Rothenberg Inc.) – which is less than the flow rate and pressure we routinely use when gassing directly from a mixed gas cylinder at approximately 10 PSI (69 kPa). We compared O_2_ and CO_2_ concentrations in each method by measuring gas concentrations with a Dräger X-am 5000 Gas Monitor fitted with XXS E O_2_ and XXS CO_2_ sensors (Table 1, Useful accessories). Four independent measurements were performed. We observed that the time to 1% oxygen was 1.6 minutes for a pre-mixed cylinder (CYL: 10 PSI, 69 kPa) and 3.8 minutes for the gas-mixing device (GM: 2 PSI, 14 kPa) (Figure 2A). Error bars (representing standard deviation) were extremely tight, demonstrating the accuracy and reproducibility of the measurements and implying that, once established for a new set of cylinders, a fixed gas time can be used for each chamber (Figure 2A, inset).

We next analyzed gas concentrations of commonly used field culturing methods, namely candle jar, pre-mixed gas, and gas mixed by our gas mixer (Figure 2B). Three independent measurements were performed (with five measurements for the Gas-mixing device) and error bars represent standard deviation. We observed that compared to ambient air with an oxygen concentration of 20.9%, the candle jar resulted in a depletion of oxygen to 16.1% and an increase in carbon dioxide levels to 3.5%, the pre-mixed gas cylinder resulted in oxygen levels of 1.03% and carbon dioxide levels of 5.0%, and the gas mixer resulted in oxygen levels of 1.18% and carbon dioxide levels of 5.0%. Our values for the candle jar were similar to those previously measured by gas chromatography: 80% N_2_, 3% CO_2_, 17% O_2_ [7].

It has been proposed that gas concentration can affect parasite phenotypes such as rosetting rates, growth rates, and drug IC_50_s. There is an extensive literature evaluating candle jars versus mixed gas as well as various kinds of flow devices [1], [14], [15], [16], [17], [18], [19], [20], [21]. While side-by-side comparisons of candle jar and mixed gas showed small or no difference in growth rate [20], [21], [22], differences in antimalarial IC_50_s have been observed. While in most studies, changes in oxygen concentration result in no statistically significant changes in the IC_50_s of chloroquine or other quinolone containing antimalarial drugs [23], [24], [25], some studies do report differences [26]. The IC_50_s of other classes of antimalarial drugs (such as antibiotics and mitochondrial inhibitors) were affected by the oxygen concentrations found in a candle jar compared to that of mixed gas (1% O_2_, 3% CO_2_) [23]. Changes in carbon dioxide concentrations have been reported to have significant effects on the IC_50_s of chloroquine [27].

These results emphasize the importance of considering the gas concentrations and culture method used when comparing field-generated drug resistance data, but also emphasizes the need for standardization of a robust, reproducible, and practical solution to *in vitro* culture in the field. The challenge of culturing the blood stage of *P. vivax* may also be due in part to an optimal oxygen tension that is different than that for *P. falciparum*. Our adjustable device will allow us to systematically test this hypothesis.

### Performance of Gas Mixer Compared to Pre-mixed Cylinder Gas

As we achieved the same oxygen and carbon dioxide concentrations as pre-mixed cylinder gas (Figure 2B), it was not necessary to test a broad range of phenotypic assays. However, with an adjustable gas mixer such as this one, the effect of varying oxygen concentrations could be tested for the same parasites in the same assays.

We first sought to evaluate our device by evaluating the functional readout of parasite growth. As this device is ideally suited for field-implementation, we tested a broad range of parasites from hardy laboratory isolates to fragile, previously uncultured clinical isolates. We found in quantitative 4-cycle growth assays, semi-quantitative long-term culture assays, and quantitative thaw comparisons, that our gas mixer was equivalent to pre-mixed gas cylinder in all assays tested.

### Side-by-side Parasite Growth Comparisons

**Figure 3:**
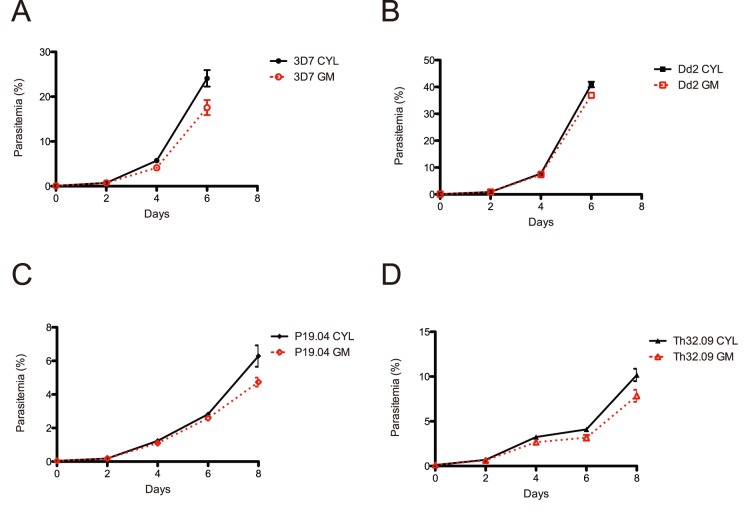
Quantitative 4-cycle growth assays. A & B. Laboratory adapted isolate 4-cycle growth rate comparisons: A. 3D7 and B. Dd2. C & D. Short-term adapted Senegalese isolate 4-cycle growth rate comparisons: C. P19.04 and D. Th32.09. Results from individual experiments are shown, conducted in triplicate, with error bars representing standard error.

We validated the gas mixer by comparing it to the gold standard of pre-mixed gas in quantitative 4-cycle growth assays. The advantage of these assays is that there is no manipulation of the cultures after the initial set up. We performed these assays with two robust laboratory adapted strains (3D7 and Dd2) (Figure 3A and 3B), as well as two short-term adapted clinical isolates from Senegal (P19.04 and Th32.09) (Figure 3C and 3D). Cultures were seeded at initial parasitemia of 0.05%, split in two dishes, and cultured either in a modular incubator chamber gassed with a pre-mixed cylinder or the gas-mixing device, with orbital shaking in the same 37 degree C incubator. Parasitemia was measured by flow cytometry after each cycle and cultures were allowed to continue until 4 cycles of growth (or until parasite crash, as was observed for 3D7 and Dd2 by cycle 4). For all strains tested, there was no difference over 3–4 cycles of growth between the pre-mixed cylinder and the gas-mixing device (Figure 3).

**Figure 4:**
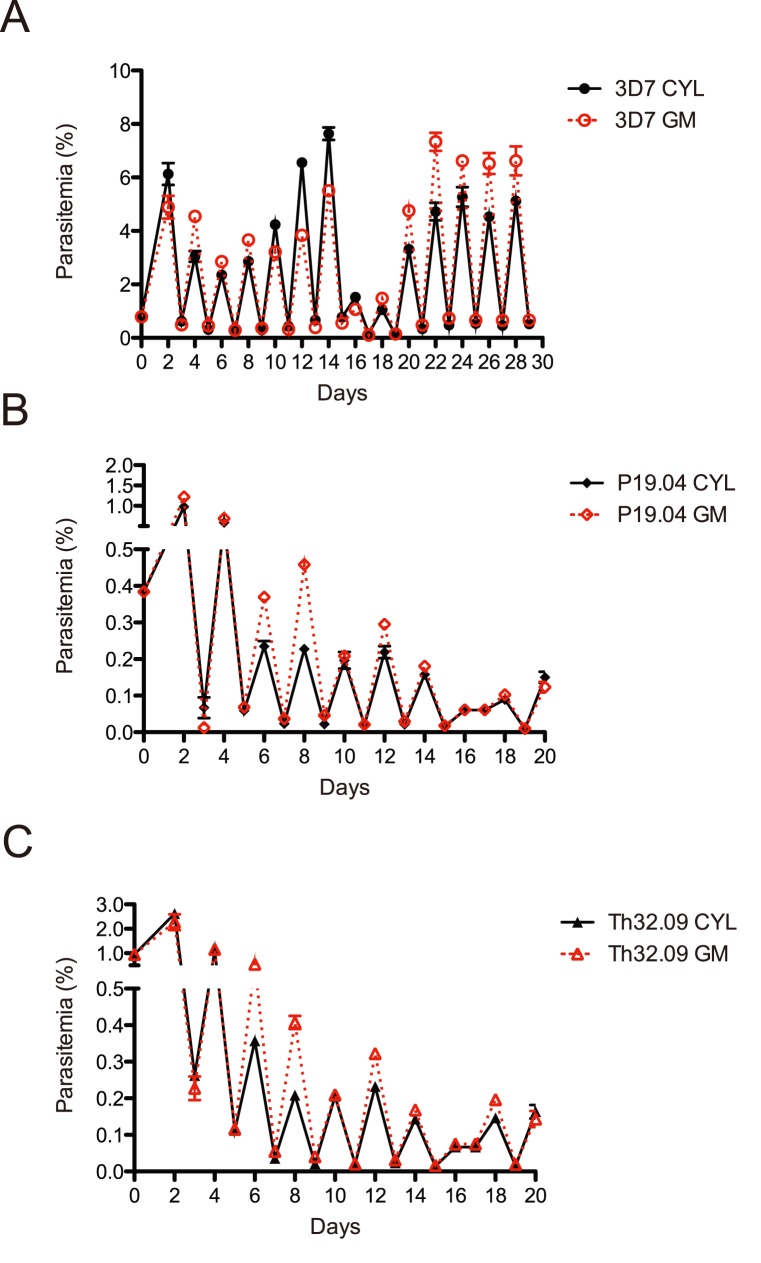
Semi-quantitative comparisons of long-term routine culture. A. Laboratory adapted (decades) isolate (3D7) routine growth comparisons. Cultures were split 1∶10 every cycle. B & C. Short term adapted (months) Senegalese isolates routine growth comparisons. Cultures were split 1∶10 every cycle, or media change only as appropriate, as indicated by action on odd days. Results from individual experiments are shown, conducted in triplicate, with error bars representing standard error.

We performed semi-quantitative comparisons of long-term culture (between 20–30 days) to validate the gas mixer over many cycles of parasite replication (Figure 4). These assays are semi-quantitative because manipulation is necessary in the long-term culture process–cultures were split 1∶10 every cycle. Cultures were seeded at initial parasitemia of 1%, split in two dishes, and cultured either in a modular incubator chamber gassed with a pre-mixed cylinder or the gas-mixing device, with orbital shaking in the same 37 degree C incubator. We tested the robust laboratory isolate 3D7 (Figure 4A) in addition to two short-term adapted isolates from Senegal (P19.04 and Th32.09) (Figure 4B and 4C). No difference was observed in the growth rates between pre-mixed cylinder and the gas-mixing device over time.

When gas concentrations were optimal (5% CO_2_, 1% O_2,_ N_2_ balance) we observed no difference in growth rates between the pre-mixed cylinder and the gas-mixing device. However, we observed a difference in growth rates when the oxygen concentration was higher in the gas-mixing device (Figure S1). For the first cycle of growth, the gas-mixing device was used at a final oxygen concentration of 5% rather than 1%, and an approximately 2.5 fold difference was observed between the cylinder and the gas-mixing device. Cultures were split 1∶10 and the oxygen concentration was decreased to 1%. At this stage in the experiment, the gas-mixing cultures were “rescued”, and subsequent growth in both methods was comparable. This experiment illustrates the importance of low oxygen concentration on robust parasite growth as well as the advantage of an adjustable gas-mixing device to test the impact of different gas concentrations on parasite phenotypes.

**Figure 5:**
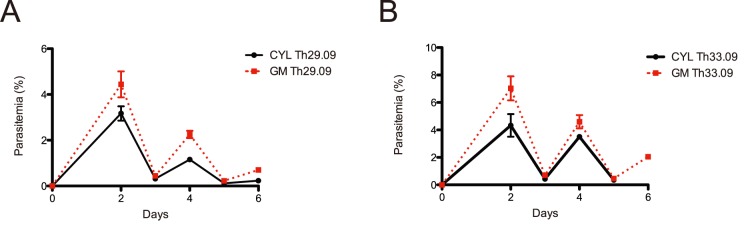
Cryopreserved field isolate recovery time. Time to positive growth from field prepared cryopreserved parasites without previous culture adaptation: A. isolate Th29.09. B. isolate Th33.09. Results from individual experiments are shown, conducted in triplicate, with error bars representing standard error.

We validated the gas mixer in the recovery of fragile, previously non-adapted cryopreserved parasites from Senegal by comparing 2 isolates: Th029.09 and Th033.09. These isolates were thawed, split in two dishes, and cultured either in a modular incubator chamber gassed with a pre-mixed cylinder or the gas-mixing device, with orbital shaking in the same 37 degree C incubator (Figure 5). The recovery times and growth rates were the same for both isolates, demonstrating that this device can be used for primary culture adaptation of *P. falciparum*.

### Field Implementation: Senegal Malaria Collection 2013

We next tested our gas-mixing device for the culture adaptation of primary isolates from Thiès, Senegal during our 2013 collection. Our goal was to perform side-by-side comparisons with a pre-mixed gas cylinder, as we did in Figures 3–5. However, as evidence of the challenges with ordering and delivery of pre-mixed gas in malaria endemic countries, our pre-mixed cylinders (ordered 10 months prior) had yet to arrive at the start of the collection. We selected parasites with greater than 0.4% parasitemia for long-term (greater than 2-cycles) *in vitro* culture adaptation (Figure S2). We successfully cultured 63 of 63 isolates for between 9 and 45 days. All cultures were growing robustly and required subculture each cycle. A subset of cultures was frozen down on collection day 28 prior to termination on day 45 due to capacity constraints with so many successful cultures.

In addition to *in vitro* culture adaptation, a number of *ex vivo* phenotypic assays were performed using our gas-mixing device. We successfully performed Erythrocyte Invasion Assays, Drug-Resistance Assays, Rosetting Assays, Variant Surface Antigen (VSA) Flow Cytometry Assays, demonstrating the utility of this device for biological experiments in malaria-endemic settings.

### Advantages of the Adjustable Gas Mixer Over Existing Alternatives

While the gas mixer demonstrated equivalent performance to the pre-mixed gas cylinder, it has a number of advantages that are especially relevant in disease endemic settings. The gas used by the gas mixer is 5–20 times less expensive than available pre-mixed alternatives, and locally available, dramatically decreasing the time to delivery from minimum of 6 months to 2 days (Table S1). These advantages are critical to performing routine, *in vitro* culture in the field and for carrying out primary research in malaria endemic countries. The cost of the gas mixer itself is $5838.00, which is approximately the cost of a single cylinder of mixed gas when ordered and delivered from outside of Africa (Table S1). While the cost difference and the supply may not dramatically affect laboratories in developed countries, these factors play a major role in culture feasibility in disease endemic countries. The device described in this study represents an accurate, affordable, and effective means of conducting robust *ex vivo* and short term *in vitro* assays in disease endemic countries.

### Further Experimental Applications

It has previously been shown that dramatic changes occur in the parasite after long term *in vitro* culture adaptation, especially in genes that mediate virulence properties [4], [5], [28], [29], [30], [31]. Having the ability to conduct robust experiments in rural, disease endemic settings, as close as possible to the *in vivo* state of the parasites within the human host represents a fundamental advance for the field and an important application for this gas-mixing device.

Additionally, this adjustable gas-mixing device provides the ability to vary experimental conditions in an extremely reproducible fashion, which may have applications for the culture of other *Plasmodium* species (such as *P. vivax*) as well as modeling gas concentrations relevant to different host niches or different malaria disease states. Such applications reach beyond the field application described here and may be useful for laboratories in non-disease endemic countries as well.

## Materials and Methods

### Ethics Statement

This study was approved by both the Institutional Review Board of the Harvard School of Public Health (CR-16330-01) and by the Ethics Committee of the Ministry of Health in Senegal (0127MSAS/DPRS/CNRES). All patient samples used in this study came from consenting uncomplicated malaria patients. Written consent was obtained from all patients, or their parents or guardians for minors, provided they could read French; for those who could not, oral consent was obtained. The patient being consented, or their parents or guardians, as well as a third party documented consent and signed consent forms were stored in a secured location. The ethics committees and IRB approved these consent procedures.

### Details and Design of 954 Flow Meter and Sensors

We designed our gas-mixing device with a number of optimizations to make it more amenable to the application of *in vitro P. falciparum* growth. First, we selected a digital flow control box with dual voltage possibility (115 V/230 V input) to facilitate its use in many countries. Secondly, we adjusted the output flow rate on the flow controllers so that the input maximum flow rate should not exceed 25 psi ∼1.75 bar (172 kPa) of pressure in the lines, and the output pressure is a maximum of 2 PSI (14 kPa) – the maximum pressure allowed by the modular incubator chamber, and a flow rate of 10 SLPM. Further, rather than a fixed flow rate box, we opted for an adjustable model which allows us to vary all gas concentrations.

We also added a homemade gas-mixing coil, post union cross, (Figure 1) to facilitate the mixing of the gas prior to entry into the modular incubator chamber. (This coil was made by wrapping copper tubing around a fire extinguisher). After complete set up, the system was confirmed to be leak free.

### Measurement of Gas Levels

Measurement of gas levels was performed using a Dräger X-am 5000 Gas Monitor (Cat. No. 4543749) fitted with XXS E O_2_ (Cat. No. 6812211) and XXS CO_2_ (Cat. No. 6810889) sensors and modified for confined space entry using a calibration cradle (Cat. No. 8318752) to permit the measurement of gas flow through PVC tubing either at incubator chamber entry or exit.

### Parasites

*P. falciparum* isolates used in this study came from several sources: long-term laboratory adapted isolates: 3D7 and Dd2; short-term culture adapted Senegalese isolates (in culture for 1 month each): P19.04, Th32.09; un-adapted cryopreserved Senegalese isolates: Th029.09, Th033.09; and *ex vivo* patient Isolates from Senegal (Th001.13–Th116.13). Infected erythrocytes were cultured in O+ human erythrocytes at 2% hematocrit in RPMI-1640 based media supplemented with 25 mM HEPES (EMD Biosciences), 2 mg/ml sodium bicarbonate, 50 µg/ml hypoxanthine, and 0.25% Albumax II (Invitrogen) and 5% human O+ serum. For long and short term adapted parasites, cultures were triple synchronized with 5% D-sorbitol prior to assay initiation at rings. Chemicals were purchased from Sigma unless otherwise specified. Cultures were monitored every 48 hours at which point gas was exchanged.

### Measurement and Comparison of Parasitemia

Quantitative 4-cycle growth assays were performed as previously described [32]. Briefly, cultures were seeded at low parasitemia (0.05%) and low hematocrit (0.25%), split into two dishes, and cultured either in a modular incubator chamber gassed with a pre-mixed cylinder (CYL) or the gas-mixing device (GM). Cultures were incubated with orbital shaking (50 rev/min), to optimize for high parasitemia and low multiplicity of infection [22].

Semi-quantitative comparisons of long-term routine culture were also performed to measure the growth differences over time. For these experiments, cultures were seeded at an initial parasitemia of 1%, split into two dishes and cultured either in a modular incubator chamber gassed with a pre-mixed cylinder (CYL) or the gas-mixing device (GM), with orbital shaking (50 rev/min), to optimize for high parasitemia and low multiplicity of infection [22]. Cultures were split 1∶10 every cycle and parasite growth and morphology were monitored by standard microscopy.

Parasitemia was quantitatively measured by SYBR Green Flow Cytometry [33] at each re-invasion cycle. While “1 cycle” is approximately 48 hours for all strains, over-long term culture the synchronicity of the culture will disintegrate resulting in a mixed-stage culture. Parasitemia measurements by flow cytometry include all stages (rings, trophs, and schizonts), culture viability is monitored by microscopy (to ensure the absence of gametocytes or pyknotic forms) and gas is changed every 48 hours.

## Supporting Information

Figure S1 **The quantitative effect of oxygen concentration on parasite growth.** The effect of oxygen concentration on parasite growth was measured for two short-term adapted field strains: P19.04 (A) and Th32.09 (B). From Day 0 to Day 2 (the first cycle of growth), the oxygen concentration was 5% whereas the pre-mixed cylinder was fixed at 1%. A 2.5-fold difference in growth was observed for both strains. All cultures were split 1∶10 and the oxygen concentration was decreased to 1% (the same as the pre-mixed cylinder). From this point onward, cultures grew equivalently. Results from individual experiments are shown, conducted in triplicate, with error bars representing standard error.
(EPS)

Figure S2 **Senegal malaria collection 2013.** Selected samples from the 2013 collection are shown, days in culture from enrollment date until final collection day. Parasites with greater than 0.4% parasitemia were *in vitro* culture adapted using gas from the gas mixer and static culture conditions. Each bar represents an individual sample with robust growth (requiring subculture) from the day of collection enrollment until termination, either by freezing down (Day 28) or culture termination (Day 45). All cultures were growing robustly at the time of termination.
(EPS)

Table S1 **Costs and logistical comparisons affecting feasibility of obtaining gas supplies from different sources.**
(DOCX)
