# Supplementary material for: An Adjustable Gas-Mixing Device to Increase Feasibility of In Vitro Culture of Plasmodium falciparum Parasites in the Field
Source: PLoS One. 2014 Mar 6;9(3):e90928. doi: 10.1371/journal.pone.0090928 (PMC3946284; doi:10.1371/journal.pone.0090928)
Supplement: Table S1 — Costs and logistical comparisons affecting feasibility of obtaining gas supplies from different sources. (DOCX) [file pone.0090928.s003.docx]

| **Gas** | **Region** | **Cost per cylinder (in USD)** | **Cost of cylinder equivalent (in USD)** | **Time**  **to delivery** | **Number required for 1 Malaria season collection ^a^** | **Cost for 1 Malaria season collection ^a^** |
| --- | --- | --- | --- | --- | --- | --- |
| 94% N_2_, 5% CO_2_, 1%O_2_ | United States | $60.00 | $60.00 | NA |  |  |
| 94% N_2_, 5% CO_2_, 1%O_2_ | France | $4,800.00 | $4,800.00 | 12 months | 2 | $9,600.00 |
| 94% N_2_, 5% CO_2_, 1%O_2_ | The Gambia | $1,000.00 | $1,000.00 | 6-10 months | 2 | $2,000.00 |
| 94% N_2_, 5% CO_2_, 1%O_2_^b^ | Mali | $718.00 | $718.00 | 6-10 months ^c^ | 2 | $1,436.00 |
| 100% N_2_ gas, medical grade, 14m^3^ | Senegal | $109.00 | $287.00 | 2 -7 days | 2 | $396.00 |
| 100% CO_2_ gas, medical grade, 28kg | Senegal | $100.00 |  | 2 -7 days | 1 |  |
| 100% O_2_ gas, medical grade, 10m^3^ | Senegal | $78.00 |  | 2 -7 days | 1 |  |

**Table S1.** Costs and logistical comparisons affecting feasibility of obtaining gas

supplies from different sources

| a - approximately 300 *ex vivo* samples, including invasion assays, drug resistance assays, culture adaptation |
| --- |
| b - 5% O_2_ compared to 1% O_2_ from other sources |
| c - gas delivery now unavailable due to political unrest in the region |
